# A hierarchical abscission program regulates reproductive allocation in *Prunus* × *yedoensis* and *Prunus sargentii*

**DOI:** 10.1093/hr/uhaf317

**Published:** 2025-11-14

**Authors:** Woo-Taek Jeon, Jeong-A Kim, Ahyeon Cheon, Shawn S Y Lee, Joohyun Kang, Jung-Min Lee, Yuree Lee

**Affiliations:** School of Biological Sciences, Seoul National University, Seoul 08826, Republic of Korea; School of Biological Sciences, Seoul National University, Seoul 08826, Republic of Korea; School of Biological Sciences, Seoul National University, Seoul 08826, Republic of Korea; Department of Plant Science, College of Agriculture and Life Science, Seoul National University, Seoul 08826, Republic of Korea; School of Biological Sciences, Seoul National University, Seoul 08826, Republic of Korea; Research Center for Plant Plasticity, Seoul National University, Seoul 08826, Republic of Korea; School of Biological Sciences, Seoul National University, Seoul 08826, Republic of Korea; School of Biological Sciences, Seoul National University, Seoul 08826, Republic of Korea; Research Center for Plant Plasticity, Seoul National University, Seoul 08826, Republic of Korea; Plant Genomics and Breeding Institute, Seoul National University, Seoul 08826, Republic of Korea

## Abstract

Organ abscission is essential for optimal reproduction, yet its regulation in perennial woody plant species is poorly understood. To investigate how abscission is spatially and temporally regulated during reproduction, we analyzed five sequential abscission events in the cherry species *Prunus* × *yedoensis* (*Cerasus* × *yedoensis*, Somei-Yoshino) and *Prunus sargentii* var. *verecunda (*Bunhong-Beot): abscission of the petals, calyces, flower pedicels, fruit pedicels, and peduncles. The abscission zone of the calyx formed *de novo* upon activation, whereas other abscission zones were pre-formed but developmentally arrested. Localized ethylene responsiveness reactivated these zones, promoting cell division, differentiation of residuum and secession cells on either side of the abscission zone, and lignin deposition in some cases. This progression was accompanied by reactive oxygen species accumulation and pH shifts. We observed species-specific differences during early floral abscission: *P. yedoensis* shed petals rapidly in a pollination-independent manner, whereas *P. sargentii* retained petals on unpollinated flowers, which later abscised with the pedicel, potentially extending the fertilization window. Both species employed a post-fertilization developmental gate via fruit pedicel abscission to selectively eliminate small, slow-growing fruits. These findings reveal that *Prunus* species coordinate a hierarchical abscission program functioning as a multilayered reproductive filter, progressively refining investment decisions to determine the final fruit set.

## Introduction

Abscission is a biological program by which entire organs—such as leaves, flowers, and fruits—are actively separated from the plant body at defined cleavage sites known as abscission zones (AZs) [1]. This evolutionarily conserved mechanism serves diverse functions across kingdoms in processes including development, reproduction, and defense [2–5]. Abscission proceeds through a series of coordinated cellular events within the AZ: the differentiation of specialized cell layers, the acquisition of competence to perceive and respond to abscission signals, and the enzymatic remodeling of the cell wall that enables cell separation and organ detachment [6].

Recent studies in *Arabidopsis thaliana* revealed an additional layer of cellular specialization within the AZ. Upon activation, AZ cells differentiate into secession cells (SECs), which form a lignin brace to confine cell wall-degrading enzymes, and residuum cells (RECs), which later acquire epidermal-like identity and deposit a protective cuticle [7–9]. Whether the differentiation of RECs and SECs is conserved across species remains to be elucidated. By contrast, lignin involvement in abscission has been documented in diverse taxa, albeit with varying roles. In pepper (*Capsicum annuum*), lignification within the AZ correlates with efficient shedding, whereas in tomato (*Solanum lycopersicum*) abscission requires the AZ to remain nonlignified, with *jointless-2* mutants failing to abscise due to lignin intrusion into the AZ [10, 11]. Grasses add further complexity, as *weedy Oryza sativa* forms a nonlignified AZ within lignified pedicel tissues, while *Brachypodium distachyon* shows clear AZ lignification associated with shedding [12]. These contrasts indicate that lignification does not operate as a universal trigger but rather plays diverse, context-dependent roles. Nonetheless, its recurrent involvement across taxa underscores lignification as a critical factor that must be precisely regulated for proper execution of abscission.

The execution of abscission is not autonomous but governed by signaling networks that coordinate cellular differentiation and cell wall remodeling. Phytohormones provide the primary regulatory framework: ethylene and abscisic acid (ABA) promote abscission, whereas auxin and cytokinin antagonize it [13, 14]. Central to this hormonal control is the INFLORESCENCE DEFICIENT IN ABSCISSION (IDA) pathway. In this pathway, IDA peptides activate the receptor-like kinases HAESA (HAE) and HAESA-LIKE2 (HSL2) together with the coreceptors SOMATIC EMBRYOGENESIS RECEPTOR-LIKE KINASEs (SERKs), triggering a mitogen activated protein kinase (MAPK) cascade that induces genes encoding cell wall-remodeling enzymes [15–20]. Ethylene directly reinforces this module by upregulating *IDA* expression, thereby linking hormonal signaling to the execution of cell separation [21].

The IDA module, first characterized in *Arabidopsis*, is highly conserved across angiosperms, from herbaceous species to woody perennials. In litchi (*Litchi chinensis*), mango (*Mangifera indica*), and citrus (*Citrus* spp.), multiple *IDA-like* genes have been identified, several of which promote abscission when expressed in *Arabidopsis* [22–24]. For example, in litchi the ethylene signaling factor LcEIL3 directly activates *LcIDL1*, and silencing of *LcMPK3 or LcMPK6* suppresses fruit abscission, while the abscission defect in *Arabidopsis* caused by simultaneous *AtMPK3/6* silencing is rescued by heterologous expression of *LcMPK3* or *LcMPK6* [25, 26]. These findings establish the IDA–MAPK cascade as a conserved core module linking phytohormones to abscission execution across annual herbs and woody fruit trees.

In addition to this phytohormone-driven transcriptional cascade, intracellular physiological cues—including reactive oxygen species (ROS) and cytosolic pH shifts—further modulate abscission by shaping the cellular microenvironment and enzymatic activity [7, 27, 28]. ROS contribute to both the spatial and temporal regulation of abscission: spatially, they promote the localized polymerization of the lignin brace within SECs [7]; temporally, the redox balance between superoxide and hydrogen peroxide, which is modulated by the secretory manganese superoxide dismutase MSD2, determines the onset of abscission [27, 29]. In parallel, the gradual cytosolic alkalinization of AZ cells is associated with both ethylene-dependent and ethylene-independent pathways, both of which are thought to regulate enzymatic activities or act as cues for gene expression [28, 30].

Given the tight coordination between abscission and developmental signaling, its functional significance extends beyond cellular execution to broader physiological contexts. In particular, abscission plays a critical role in plant reproduction, by removing floral organs that have completed their function after fertilization, as well as by enabling seed dispersal at later stages [31]. Much of our current molecular understanding of abscission comes from studies in *Arabidopsis*, which have identified key regulators and signaling pathways. However, its self-fertilizing nature results in relatively simple floral abscission dynamics that are largely uncoupled from fertilization outcomes, limiting its utility for investigating how abscission is regulated in response to reproductive success, such as whether fertilization or embryo development has occurred. In addition, its production of dry, silique-type fruits restricts its relevance for understanding abscission during the development or detachment of fleshy fruits. Although recent studies in crops such as rice and tomato have expanded the molecular framework of abscission [32, 33], the physiological roles of abscission in shaping reproductive efficiency remain underexplored, particularly in perennial or cross-pollinating species with more complex reproductive strategies.

To overcome these limitations, the genus *Prunus* offers a valuable system that combines reproductive complexity with broad biological relevance. It includes both economically important fruit crops and ornamental species, such as peaches (*Prunus persica*), apricots (*Prunus armeniaca*), almonds (*Prunus dulcis*), and cherries (*Prunus avium* and others) [34, 35]. Among these, ornamental cherries are particularly notable for their profuse flower production and strong reliance on cross-pollination, a combination that necessitates selective regulation over reproductive investment [36]. Indeed, although profuse flowering increases the likelihood of successful fertilization, it may also result in more potential fruits than the plant is able to physiologically support. Limited nutrient availability and developmental capacity prevent all flowers from maturing into fruits. In this context, abscission likely serves as a developmental gate, modulating reproductive output in response to both fertilization success and internal resource constraints. These features make the genus *Prunus* a compelling system in which to investigate how plants coordinate organ shedding with reproductive and physiological cues.

Despite this potential, relatively little is known about how abscission is regulated across developmental stages in *Prunus*, particularly during early phases of reproduction. Most existing studies in *Prunus* have focused on fruit maturation and detachment [37–40]. These studies showed that fruit detachment can occur either at the fruit–pedicel junction or at the pedicel–peduncle (PP) junction, and that the site of separation is developmentally regulated. Specifically, abscission tends to occur at the PP junction during early stages of fruit development, whereas it typically takes place at the fruit–pedicel junction in more mature fruits [37]. In *P. avium*, early activation of abscission at the PP junction varies among individuals and is correlated with higher rates of embryo abortion [40], suggesting that abscission may function as a selective filter to eliminate reproductively unsuccessful fruits. However, how earlier abscission events, particularly those involving floral organs, are developmentally coordinated with reproductive status remains poorly understood.

In this study, we investigated how *P. yedoensis* and *P. sargentii*, two ornamental cherry species, employ sequential and hierarchical abscission programs to regulate reproductive output. We show that *P. yedoensis* exhibits pollination-independent petal shedding, but subsequent abscission events of the calyx, flower pedicel, and fruit pedicel are selectively activated based on fertilization outcome and fruit development. In contrast, *P. sargentii* retains petals on unfertilized flowers and displays whole-flower abscission, revealing a distinct strategy for early reproductive filtering. Anatomical analysis further uncovered species-specific differences in lignin deposition and timing of AZ activation.

Together, our findings show that abscission operates as a layered developmental filter that integrates developmental cues with reproductive outcomes to determine the fate of floral and fruit structures. This framework broadens the conceptual understanding of abscission beyond terminal detachment, offering new insights into its role as a key regulatory filter in reproductive optimization.

## Results

### Temporal and developmental coordination of sequential floral abscission in *P. yedoensis* and *P. sargentii*

**Figure 1:**
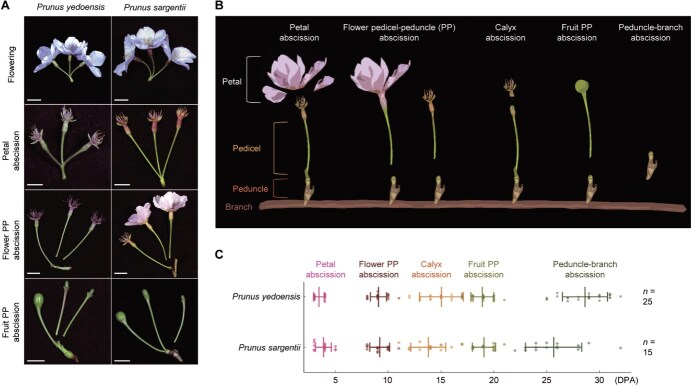
Phenotypic characterization of five distinct abscission types in *Prunus* × *yedoensis* and *P. sargentii*. (A) Representative images of flowering and each abscission type under open-field conditions. Scale bars, 1 cm. (B) Diagram depicting the five types of abscission events in two *Prunus* species: petal abscission, flower PP abscission, calyx abscission, fruit PP abscission, and peduncle-branch abscission. (C) Timing of the five abscission types in the two *Prunus* species. Data are presented as mean ± standard deviation (SD), with individual data points shown as distinct symbols. Quantification was performed under open-field conditions: for *P. yedoensis*, *n* = 25 branches from five independent trees were analyzed; for *P. sargentii*, *n* = 15 branches from three independent trees were analyzed. Data were collected in 2025.

To investigate how abscission is temporally and developmentally coordinated with reproduction in *Prunus*, we monitored the timing and sequence of abscission events in *P. yedoensis* and *P. sargentii* under open-field conditions. Each inflorescence typically bears two to four flowers, each on an individual pedicel attached to a common peduncle, providing a clear framework for staging and comparing abscission events (Fig. 1A).

Consistent with previous reports in other *Prunus* species [37, 40], both *P. yedoensis* and *P. sargentii* exhibited a sequential pattern of petal and fruit PP abscissions. Petal abscission was the earliest event, occurring ~3–4 days post-anthesis (DPA) (Fig. 1B and C). Fruit PP abscission occurred much later, at 19 DPA, when the pedicel bearing a developing fruit detached from the peduncle, typically only after the fruit had reached a defined developmental stage.

Between these two established events, we identified a previously unrecognized third stage, which we term *flower PP abscission* (Fig. 1B). This event occurred at the same anatomical junction as fruit PP abscission but was developmentally distinct, involving pedicels of unfertilized or aborted flowers at ~9 DPA.

In flowers that did not undergo flower PP abscission and remained attached, calyx abscission occurred at 14–15 DPA, marked by detachment of the sepal whorl surrounding the floral organs (Fig. 1B and C). Fruit PP abscission then proceeded at ~19 DPA, and peduncle abscission was the terminal event, taking place only after all pedicels had been shed—around 29 DPA in *P. yedoensis* and 26 DPA in *P. sargentii*.

Together, these observations define a conserved abscission sequence—petal, flower PP, calyx, fruit PP, and peduncle–branch—while also revealing a new intermediate event (flower PP abscission) that directly links floral fate to reproductive outcome. Despite this overall conservation, the developmental timing of flower PP abscission varied between the two species: in *P. yedoensis*, flower pedicel detachment typically followed petal shedding, whereas in *P. sargentii*, it frequently occurred while the petals were still attached (Fig. 1A). These contrasts suggest that, although the abscission sequence is conserved, the regulatory thresholds coordinating abscission timing differ between the two species.

### Conserved role of PyIDA in regulating abscission in *P. yedoensis*

**Figure 2:**
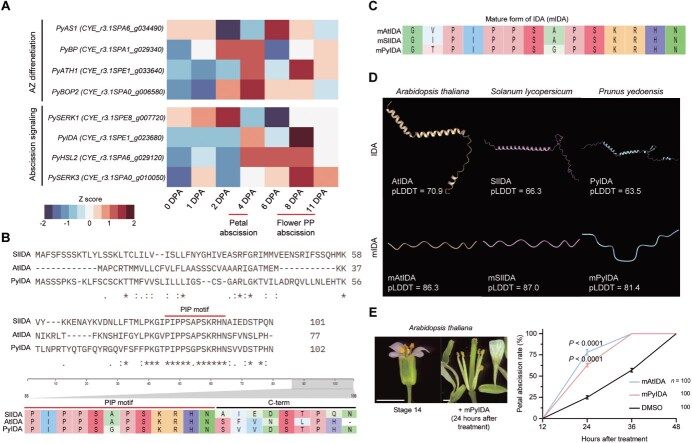
Conserved role of PyIDA in regulating abscission in *P. yedoensis*. (A) Heatmap of AZ differentiation and abscission signaling-related genes across a developmental time series from RNA-seq. (B, C) Multiple sequence alignments of full-length IDA peptides (B) and their mature form (mIDA) (C) from *S. lycopersicum* (Sl), *A. thaliana* (At), and *P. yedoensis* (Py). (D) Predicted protein structures of IDA and mIDA with confidence values shown as pLDDT scores. (E) Stage 14 *Arabidopsis* flowers treated with DMSO, mAtIDA, or mPyIDA. The proportion of flowers exhibiting petal abscission was scored at 12-h intervals (*n* = 100 flowers), with representative images shown. Differences between peptide- and DMSO-treated groups were evaluated using Welch’s *t*-tests. Scale bars, 500 μm.

To explore the molecular basis of abscission in *P. yedoensis*, we analyzed publicly available time-series RNA-seq data from whole flowers spanning flowering, petal abscission (~4 DPA), and flower PP abscission (~8–11 DPA) (Tables S1–S4) [41]. Genes functionally validated in *Arabidopsis* as regulators of AZ differentiation and abscission signaling were upregulated in *P. yedoensis* during both petal and flower PP abscission events (Fig. 2A). In addition, transcripts associated with major phytohormone pathways showed dynamic stage-specific induction (Fig. S1). These patterns indicate that canonical abscission regulators and hormone-related genes are transcriptionally activated during multiple abscission processes, raising the possibility that core components of the abscission program identified in *Arabidopsis* may also be conserved in *P. yedoensis*.

Among these genes, *PyIDA* (CYE_r3.1SPE1_g023680) exhibited a dual-peak expression pattern at 4 and 8 DPA, coinciding with the timing of petal and flower PP abscission (Fig. 2A). To examine its sequence conservation, we compared *PyIDA* peptides with IDA and IDA-like (IDL) orthologs from other species. Maximum-likelihood (ML) phylogenetic analysis grouped *PyIDA* with functionally validated IDA/IDLs (Fig. S2A, Table S5). Sequence comparison of the processed mature form of IDA (mIDA) showed that the C-terminal PIP motif (defined by the consensus sequence Pv/iPPSa/gPSk/rk/rHN), previously reported as a conserved feature among diverse plant species [15], was broadly conserved; however, the sixth residue was glycine (G) instead of alanine (A), as found in *Arabidopsis* and *S. lycopersicum* (Fig. 2B and C, Table S6). This substitution was observed in all analyzed species except *Arabidopsis* and tomato (Fig. S2B). Notably, *PyIDA* was identical in its mature sequence to *LcIDL1* (Fig. S2B), which has been functionally validated [22].

To further investigate signaling components, we analyzed the predicted structures of the IDA peptide and its putative receptors in *P. yedoensis*. Although HAE and its coreceptors SERK 2/4/5 homologs were not detected in the available datasets (Table S3), structural predictions for HSL2, SERK1, and SERK3 indicated highly similar conformations, consistent with their proposed roles in abscission signaling (Fig. S2C, Table S7) [16, 19]. Predicted mIDA structure revealed a distinct protrusion in the central region (Fig. 2D, Table S7), a feature also observed in *LcIDL1* and in *GmIDA1a/b* [22, 42], suggesting that this structural characteristic is broadly conserved among IDA peptides (Fig. S2D).

Finally, we tested functional activity by applying synthetic mAtIDA and mPyIDA peptides to Stage 14 *Arabidopsis* flowers, corresponding to 0 DPA and prior to abscission onset [43]. Both peptides triggered precocious abscission, demonstrating that PyIDA possesses functional activity comparable to AtIDA (Fig. 2E).

### Petal abscission in *P. yedoensis* is developmentally timed and independent of pollination

Although IDA-mediated signaling is conserved in *P. yedoensis*, this conservation does not explain the species-specific variation in abscission timing observed between *P. yedoensis* and *P. sargentii* (Fig. 1A). To investigate the mechanisms underlying this species-specific coordination, we first focused on *P. yedoensis*. A striking feature of this species is its rapid, synchronous petal shedding shortly after anthesis. Given that floral structures primarily function to facilitate pollination [44, 45], and that *Prunus* species depend on insect-mediated cross-pollination [46–49], this pattern raised a key question: does the rapid and synchronized petal abscission seen in *P. yedoensis* reflect high pollination efficiency?

**Figure 3:**
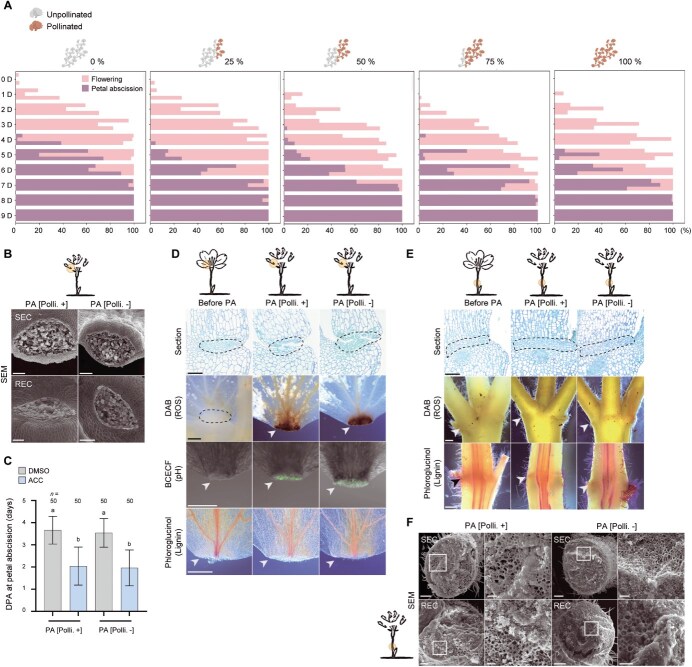
Petal abscission occurs independently of fertilization, while PP abscission is suppressed in *P. yedoensis*. (A) Temporal pattern of petal abscission in response to pollination. For each tree, the proportion of flowers that had undergone anthesis was recorded, and cross-pollination was performed on 0%, 25%, 50%, 75%, or 100% of all flowers per tree at 0–1 DPA. Daily percentages of petal abscission are shown for each group; each horizontal bar denotes an individual tree (*n* = 3 trees, greenhouse conditions). (B) Representative scanning electron micrographs showing RECs and SECs comprising the petal AZ, following manual separation at the time of petal abscission (PA), with pollination (PA [Polli. +]) or without pollination (PA [Polli. −]) (*n* = 5 flowers). (C) Time to petal abscission following treatment. Pollinated and nonpollinated flowers were treated with either DMSO or 100 μM ACC. Pollination was defined as 0 DPA, and treatments were applied at 1 DPA (greenhouse conditions). Data are shown as mean ± SD. Statistical analysis was performed using one-way analysis of variance (ANOVA) with Dunn’s *post hoc* correction (*n* = 50 flowers). Different letters above the bars indicate statistically significant differences between groups at *P* < 0.05. (D, E) Representative images of petal AZs (D) and flower PP AZs (E) obtained from sectioning and histochemical staining. Dashed lines and arrowheads indicate the location of the AZ (*n* = 5 for section images; *n* = 21 for all other staining experiments). (F) Representative scanning electron micrographs showing RECs and SECs in the PP AZ after manual separation during the PA [Polli. +] and PA [Polli. −]. White squares indicate the regions shown at higher magnification at right (*n* = 5). Scale bars, 100 μm (B); 50 μm (D and E, higher magnification images in F); 500 μm (DAB, phloroglucinol*–*HCl, and BCECF images in D and E); 200 μm (main images in F).

To address this question, we hand-pollinated flowers at defined levels (0%, 25%, 50%, 75%, or 100% of all flowers per tree) and monitored petal abscission under greenhouse conditions. Contrary to the common assumption that petal retention is linked to pollination success, flowers of *P. yedoensis* uniformly shed their petals even in the complete absence of pollination (Fig. 3A), suggesting that petal abscission is developmentally programmed. Pollinated and nonpollinated flowers had identical petal shedding kinetics (Fig. S3), confirming that petal abscission is triggered independently of pollination and follows an intrinsic schedule initiated after anthesis.

Scanning electron microscopy (SEM) analysis supported this conclusion. Indeed, we observed clean separation across the AZ regardless of pollination status. Petals manually removed at the time of natural abscission showed uniformly smooth fracture surfaces in both SECs and RECs, with no sign of torn or collapsed tissue (Fig. 3B), indicating that cell separation proceeds cleanly across the AZ via programmed cell wall remodeling, independently of pollination. Application of the ethylene precursor 1-Aminocyclopropane-1-carboxylate (ACC) accelerated petal abscission (Fig. 3C and Fig. S4A), demonstrating that the petal AZ is developmentally primed and fully responsive to ethylene*,* a key hormonal regulator of abscission [50, 51]*,* at this stage. At the cellular level, *Arabidopsis* employs spatially localized ROS accumulation to regulate the timing and pattern of abscission [7, 27]. Similarly, in *P. yedoensis*, ROS accumulated in the AZ coinciding with the onset of abscission (Fig. 3D), suggesting a conserved mechanism. Beyond a marker of timing, ROS may trigger downstream events such as localized alkalinization, which modulates the activity of cell wall-remodeling enzymes [28]. Indeed, imaging with the pH-sensitive fluorescent dye BCECF (2′,7′-bis(2-carboxyethyl)-5-(and-6)-carboxyfluorescein) revealed a marked increase in fluorescence in the AZ during abscission (Fig. 3D), indicating localized alkalinization. Together, these findings suggest that ROS and pH act in concert to regulate the spatial and temporal progression of abscission. Unlike *Arabidopsis*, which uses lignin deposition to demarcate the AZ boundary [7], *P. yedoensis* exhibited no lignin accumulation in the petal AZ (Fig. 3D). This finding suggests that pH-mediated enzyme modulation, rather than lignin-based compartmentalization, contributes to the spatial control of abscission in this species.

In contrast, the PP AZ remained inactive at this stage. Anatomical analysis revealed that the PP AZ was already structurally differentiated when petal abscission occurred (Fig. 3E). However, SEM analysis of manually detached pedicels showed torn and irregular fracture surfaces (Fig. 3F), indicating that abscission had not yet been activated. Application of the ACC also failed to induce pedicel abscission (Fig. S4B), suggesting that the AZ was not yet ethylene responsive. In support of this idea, we detected no ROS accumulation or lignin deposition in the PP AZ at this stage (Fig. 3E). These findings highlight a clear developmental separation between different types of AZs: while the petal AZ is actively executing abscission, the anatomically formed PP AZ remains dormant until later stages, underscoring the stage-specific activation of abscission programs in *P. yedoensis*.

### The divergent abscission paths of the calyx and pedicel are shaped by fruit development in *P. yedoensis*

**Figure 4:**
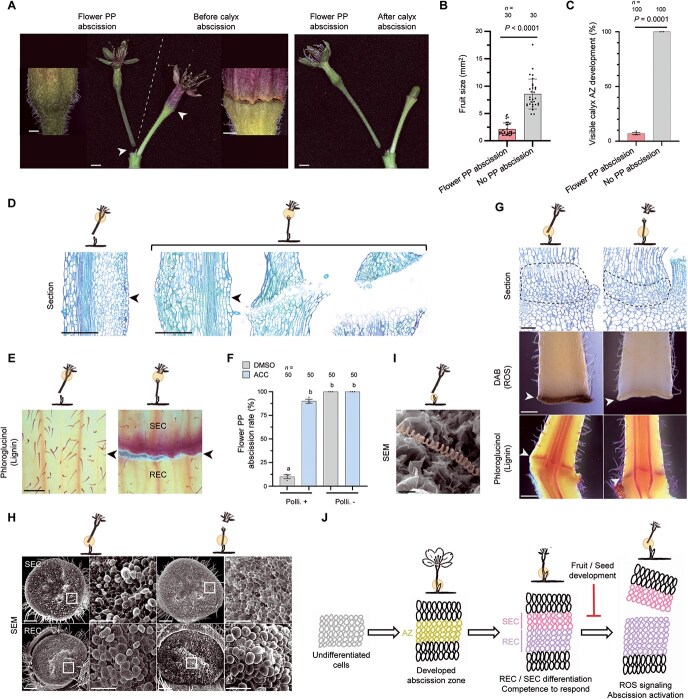
Reproductive success determines abscission type between flower PP and calyx in *P. yedoensis*. (A) Representative images of flower PP abscission and calyx abscission occurring on the same peduncle, insets showing higher magnification views of the calyx region. Arrowheads indicate the location of the AZ. (B) Fruit size at the time of flower PP abscission compared with flowers that did not undergo flower PP abscission. Samples were collected at 9–11 DPA under open-field conditions. Each dot represents one flower; data are shown as mean ± SD. Statistical analysis was performed using the Mann–Whitney test (*n* = 30 flowers). (C) Proportion of flowers developing a calyx AZ depending on whether flower PP abscission occurred (open-field conditions). Observations were made at 9–11 DPA for PP abscission and up to 20 DPA for non-abscised flowers. Data are means ± SD (*n* = 100 flowers). Welch’s *t*-test was used for statistical analysis. (D, E) Representative sections (D) and phloroglucinol–HCl staining (E) images of the calyx AZ from flowers undergoing either flower PP abscission or calyx abscission. Arrowheads indicate the expected location of the calyx AZ. Samples were collected at 13–15 DPA under open-field conditions (*n* = 5 for sections; *n* = 12 for staining). (F) Flower PP abscission rate following treatment. Pollinated and nonpollinated flowers were treated with either DMSO or 100 μM ACC at 5 DPA under greenhouse conditions. Abscission was scored at 15 DPA. Data are shown as mean ± SD (*n* = 50 flowers). Statistical analysis was performed using one-way ANOVA with Dunn’s *post hoc* test. Different letters above the bars indicate significant differences at *P* < 0.05. (G) Representative sections and histochemical staining of the flower PP AZ from flowers undergoing either flower PP abscission or calyx abscission. Dashed lines and arrowheads indicate the flower PP AZ. Samples were collected at 13–15 DPA under open-field conditions (*n* = 5 for sections; *n* = 12 for staining). (H) Representative scanning electron micrographs showing RECs and SECs in the PP AZ after manual separation at the time of flower PP abscission or calyx abscission with higher magnification views (*n* = 5). (I) Representative scanning electron micrographs of RECs from flower PP abscission, highlighting exposed spiral vessels in orange pseudo color (*n* = 5). (J) Diagram of flower PP AZ development and activation. The AZ forms during anthesis and differentiates into RECs and SECs around petal abscission, accompanied by lignin brace formation and acquisition of ethylene responsiveness (competency). Abscission is then triggered by ROS signaling in the SECs, but when fruit and seed development succeed, this step is bypassed and calyx abscission occurs instead. Scale bars, 10 mm (main images in A), 1 mm (higher magnification images in A), 500 μm (D, E, DAB and phloroglucinol images in G), 50 μm (sections in G and higher magnification images in H), 200 μm (H), 20 μm (I).

Pollination-independent petal abscission leads to the retention of many unfertilized flowers, prompting the question of how the plants eliminate these nonproductive remnants. Because these flowers, which retain reproductive organs and pedicels, offer no reproductive advantage, we hypothesized that flower PP abscission serves as clearance mechanism (Fig. 4A). Supporting this idea, the fruits from flowers that underwent flower PP abscission were significantly smaller than those that remained attached (Fig. 4B), indicating that flower PP abscission preferentially removes low-output flowers.

Notably, in flowers that remained attached, we observed an alternative abscission event at the calyx, the whorl of sepals enclosing the floral organs during early development (Fig. 4A and B). By contrast, flowers that had undergone flower PP abscission showed no calyx abscission (Fig. 4C), and in these cases there was no boundary separating the calyx from the pedicel (Fig. 4D). Unlike the PP AZ, which is pre-formed but developmentally dormant, the calyx AZ appears to be absent until later stages (≥13 DPA). Only flowers that escaped flower PP abscission developed a *de novo* AZ at the base of the calyx, comprising three to four layers of narrow cells (Fig. 4D). This zone displayed hallmark features of AZ activation: REC expansion, separation from SECs, and progressive REC enlargement. Moreover, lignin deposition occurred specifically in the SEC layer of the calyx AZ (Fig. 4E), echoing the situation in *Arabidopsis*, in which lignin functions as an apoplastic brace to spatially constrain cell wall degradation [7].

Whereas the calyx AZ arises only after fertilization, the PP AZ is already anatomically established by anthesis but remains ethylene-insensitive during petal shedding (Fig. 3E and Fig. S4). Flower PP abscission occurred selectively among flowers on the same peduncle, indicating localized regulatory control. To clarify whether this selectivity arises from differences in ethylene competence, signaling input, or structural inhibition, we compared ethylene responsiveness between pollinated and nonpollinated flowers. Abscission was markedly less frequent in pollinated flowers but occurred frequently in nonpollinated ones (Fig. 4F). However, ACC treatment restored abscission in pollinated flowers to levels comparable to that seen in nonpollinated controls, ruling out structural constraints or differential competence. These findings suggest that selective PP abscission is governed primarily by signaling differences rather than anatomical or phytohormonal readiness.

At this stage, lignin began to accumulate in the SEC of the PP AZ, a feature absent from earlier stages, indicating structural maturation (Fig. 4G). The number of AZ cell layers also increased, especially within the REC region of abscising pedicels, suggesting enhanced tissue readiness. As in *Arabidopsis* [7], lignin deposition preceded organ separation and occurred irrespective of whether abscission ultimately took place. SEM analysis of manually separated pedicels further supported this notion, revealing smooth fracture surfaces and lignified xylem strands as the final physical link in both abscised and retained pedicels (Fig. 4H and I). These observations confirm that the PP AZ is structurally delineated and primed for separation, regardless of whether abscission is ultimately executed.

Notably, we observed a more pronounced increase in AZ cell layers in pedicels undergoing PP abscission, especially within the REC region, indicating enhanced maturation associated with activation (Fig. 4G). In addition, we detected ROS accumulation only in abscising pedicels (Fig. 4G), suggesting that ROS function as a terminal trigger for abscission. These findings suggest that the PP AZ progressively acquires structural readiness and ethylene responsiveness regardless of fertilization outcome, but undergoes further maturation upon seed abortion, including ROS activation, to initiate abscission (Fig. 4J).

Together, these results demonstrate that calyx and flower PP abscissions follow distinct temporal and regulatory trajectories. Calyx abscission is induced post-fertilization in coordination with fruit development, whereas pedicel abscission serves as a selective mechanism for the removal of nonproductive flowers, integrating structural maturation, ethylene sensitivity, and ROS signaling.

### A post-fertilization abscission controls fruit number in *P. yedoensis*

Flower PP abscission efficiently eliminates unfertilized flowers at early stages, but whether all fertilized flowers are maintained through fruit development remains unclear. Insect-mediated pollination is inherently variable. Although flower overproduction helps ensure reproductive success, excessive fruit retention under high pollination success could overwhelm resource availability and compromise reproductive efficiency.

**Figure 5:**
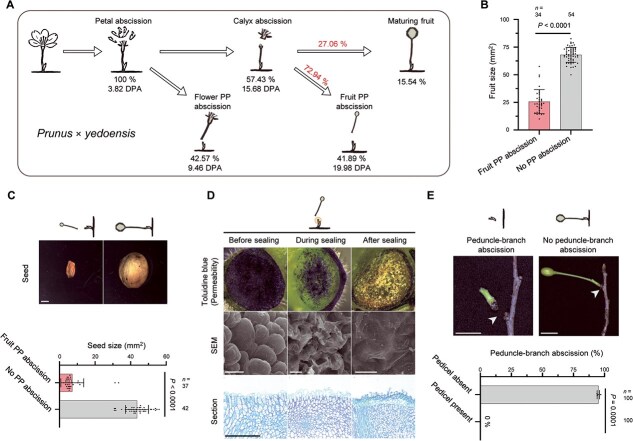
Reproductive success influences subsequent abscission types in *Prunus yedoensis*. (A) Diagram showing the timing and frequency of each abscission type. Percentages below images indicate absolute proportion relative to the total number of flowers (or fruits), and the percentages above arrows indicate relative proportions. Data are collected in 2025. (B) Fruit size at the time of fruit PP abscission in abscised versus retained fruits. Sampling was conducted at 19–22 DPA, corresponding to the period of fruit PP abscission. Each dot represents one fruit; data are shwon as mean ± SD (*n* = 54 fruits). Statistical analysis was performed using the Mann–Whitney test. (C) Seeds at the time of fruit PP abscission. Representative images (top) and seed size (bottom) from abscised versus retained fruits. Sampling was conducted at 19–22 DPA. Data are shown as mean ± SD (*n* > 36 seeds). Statistical analysis was performed using the Mann–Whitney test. (D) Development of the protective layer in RECs of the PP AZ. Representative images of toluidine blue staining, sections, and scanning electron micrographs illustrating the development of the protective layer in RECs of the PP AZ (*n* = 10 for toluidine blue staining; *n* = 5 for sections and SEM). (E) Peduncle–branch abscission. Representative images (top) and frequency (bottom). Arrowheads indicate the flower peduncle–branch AZ. Data are shown as mean ± SD (*n* = 100). All experiments were conducted under open-field conditions. Statistical analysis was performed using Welch’s *t*-test. Scale bars, 2 mm (C), 500 μm (toluidine blue and sections in D), 20 μm (SEM in D), 1 cm (E).

We hypothesized that *P. yedoensis* employs a secondary filtering mechanism after fertilization to regulate final fruit load. To test this hypothesis, we tracked the fate of flowers and developing fruits across stages. Remarkably, a large proportion of fruit PP abscission occurred after fruit development had already initiated, accounting for 41.9% of all flowers and 73.0% of calyx-abscised fruits (Figs 1A and 5A). This post-fertilization abscission was strongly associated with smaller fruit size: abscised fruits were on average ~38.5% as large as retained fruits (Fig. 5B), and their seeds were ~16.7% as large as those in retained fruits (Fig. 5C). These findings indicate that *P. yedoensis* employs post-fertilization fruit selection via fruit PP abscission to eliminate low-quality fruits, thereby optimizing resource allocation and enhancing reproductive efficiency. This filtering may reflect intrinsic fruit quality, interfruit competition, or both, acting as a developmental gate.

Given the high frequency of post-fertilization abscission, maintaining tissue integrity at the exposed site is essential. To examine how *P. yedoensis* achieves this, we assessed surface permeability using toluidine blue staining. We observed a gradual decline in staining intensity over time, indicating that a protective barrier forms post-abscission (Fig. 5D). Unlike in *Arabidopsis*, where RECs transition into epidermal-like cells and develop a cuticle [7], *P. yedoensis* formed a yellowish, translucent material that spread over the abscised surface. SEM analysis revealed that this material originates from RECs and flows across cell boundaries (Fig. 5D). Longitudinal sections showed dynamic changes in the RECs, including outer cell expansion, inner cell divisions, and eventual collapse of the outermost layer (Fig. 5D). These observations suggest a multilayered, species-specific sealing mechanism involving coordinated cellular remodeling.

Once all associated pedicels had abscised, the peduncle itself was also shed (Fig. 1B and C). Specifically, 95% of peduncles abscised when all pedicels were gone, whereas those retaining even a single pedicel remained intact (Fig. 5E). As with other abscission events, we detected evidence of ROS accumulation, as well as the formation of a protective layer on branch-side RECs (Fig. S6). Collectively, these findings reveal that *P. yedoensis* orchestrates a sequential and hierarchical abscission program, from the early removal of unfertilized flowers, to the post-fertilization elimination of underdeveloped fruits, and ultimately to the shedding of spent peduncles. This multilayered strategy ensures efficient reproductive investment and resource optimization throughout its reproductive phase.

### Species-specific adjustment of hierarchical abscission in *P. sargentii*

**Figure 6:**
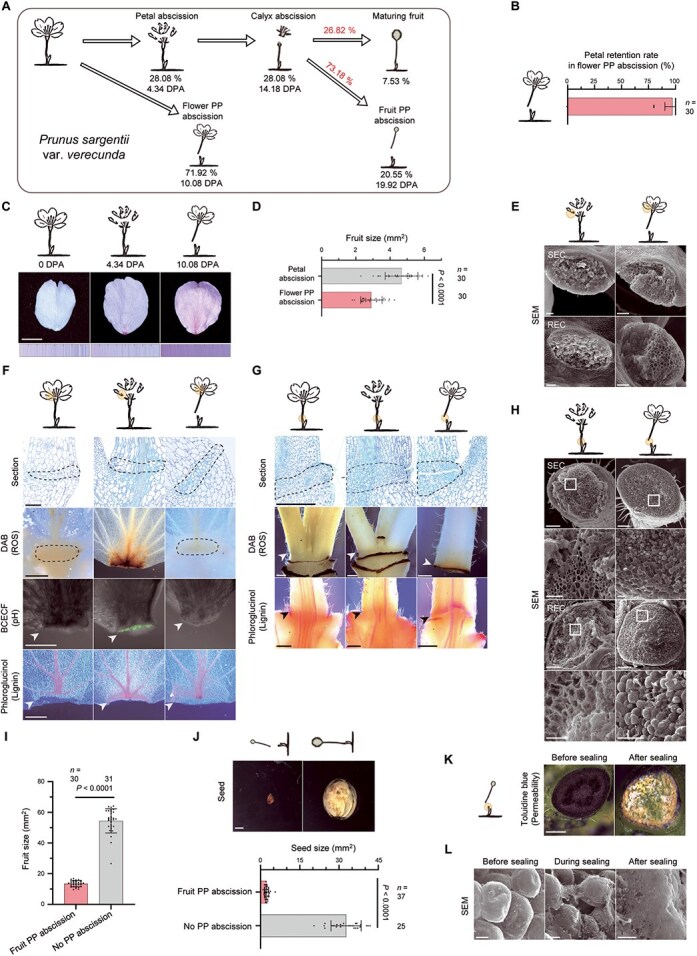
Choice between petal and flower pedicel abscission types in *P. sargentii* is guided by reproductive success. (A) Diagram showing the timing and frequency of each abscission type. Percentages below images indicate absolute proportion relative to the total number of flowers (or fruits), and the percentages above the arrows indicate relative proportions. Data are collected in 2025. (B) Number of petals remaining at the time of flower PP abscission. Each dot represents one flower; data are shown as mean ± SD (*n* = 30 flowers). (C) Transition of petal color from anthesis to petal abscission and flower PP abscission. Representative images are shown with color palettes based on 400 measurements (*n* = 20 flowers). (D) Fruit size at the time of flower PP abscission in flowers undergoing petal versus flower PP abscission. Each dot represents one fruit; data are shown as mean ± SD (*n* = 30 fruits). Statistical analysis was performed using the Welch’s *t*-test. (E) Representative scanning electron micrographs showing RECs and SECs of the petal AZ after manual separation at the time of petal and flower PP abscission (*n* = 5). (F, G) Representative sections and histochemical staining of the petal AZ (F) and the PP AZ (G) at anthesis, petal abscission, and flower PP abscission. Dashed lines and arrowheads indicate the AZ (*n* = 5 for sections; *n* ≥ 12 for staining). (H) Scanning electron micrographs of RECs and SECs in the PP AZ following manual separation at petal or flower PP abscission with higher magnification views shown below (*n* = 5). (I) Fruit size at the time of fruit PP abscission in abscised versus retained fruits. Each dot represents one fruit; data are shown as mean ± SD (*n* > 29 fruits). Statistical analysis was performed using the Mann–Whitney test. (J) Seeds at the time of fruit PP abscission. Representative images (top) and seed size (bottom) from abscised versus retained fruits. Each dot represents one seed; data are shown as mean ± SD (*n* > 24 seeds). Statistical analysis was performed using the Mann–Whitney test. (K) Toluidine blue staining of PP AZs showing development of the protective layer in RECs before (fruit PP abscission) and after sealing (6 days later) (*n* = 15). (L) Scanning electron micrographs of RECs in the PP AZ illustrating protective layer development before (fruit PP abscission), during (3 days later), and after sealing (6 days later) (*n* = 5). All experiments were conducted under open-field conditions. Scale bars, 0.5 cm (C), 100 μm (E), 50 μm (sections in F, higher magnification images in H), 500 μm (DAB, BCECF, phloroglucinol*–*HCl images in F, G, K), 200 μm (main images in H), 2 mm (J), 10 μm (L).

To determine whether the hierarchical sequence of abscission sequence observed in *P. yedoensis* represented a species-specific adaptation or a general reproductive strategy, we extended our analysis to the closely related species *P. sargentii*. When abscission events were tracked at the level of individual peduncles, *P. sargentii* displayed a similarly ordered progression: petal abscission occurred first, followed by flower PP abscission, calyx abscission, and finally fruit PP abscission (Fig. 6A). This stepwise filtering resulted in final fruit set accounting for <8% of the initial number of flowers (Fig. 6A). Notably, both species exhibited strikingly similar rates of fruit PP abscission among calyx-abscised fruits (*P. yedoensis*: 72.9%; *P. sargentii*: 73.2%), suggesting a conserved mechanism regulating final fruit load.

However, key differences emerged during the flower PP abscission stage. In *P. yedoensis*, petal abscission consistently preceded flower PP abscission, such that petals were rarely present when pedicel detachment occurred (Fig. S7). By contrast, *P. sargentii* almost always underwent flower PP abscission with the petals still attached (Fig. 6B).

Notably, petal color gradually shifted from white at anthesis to a mauve pink hue, becoming most prominent by the time of flower PP abscission (Fig. 6C, Fig. S8, and Table S8). Floral color change, defined as age- or pollination-dependent shifts in petal pigmentation, is a widespread strategy in angiosperms to enhance reproductive efficiency [52–54]. These observations suggest that successful fertilization accelerates petal abscission, whereas unfertilized flowers retain petals longer before being discarded via pedicel abscission. Consistent with this idea, petal abscission was associated with well-developed fruits, whereas flower PP abscission frequently occurred in smaller, underdeveloped fruits (Fig. 6D). The higher frequency of flower PP abscission in *P. sargentii* (71.9%) compared to *P. yedoensis* (42.6%) (Figs 5A and 6A) supports the idea that *P. sargentii*, potentially due to lower fertilization efficiency, extends the fertilization window by delaying petal shedding. Nevertheless, other factors such as nutrient limitation or stress responses may also contribute to this pattern.

Forcible removal of petals at the time of flower PP abscission resulted in torn and irregular surfaces, as revealed by SEM, indicating that abscission had not yet been activated (Fig. 6E). Histological analysis supported this notion: although the petal AZ was pre-formed at anthesis, we observed ROS accumulation and cellular alkalinization only when petal abscission was actively proceeding (Fig. 6F). Unlike *P. yedoensis*, which lacks lignin deposition at the petal AZ, *P. sargentii* formed a lignin brace in the SECs only during active petal abscission, suggesting that lignification is part of a post-fertilization activation program. We observed a similar pattern at the flower PP AZ, where both ROS accumulation and lignin deposition coincided with abscission onset (Fig. 6G), as confirmed by SEM (Fig. 6H).

Following fertilization, the subsequent steps appeared highly conserved between the two *Prunus* species. Calyx abscission, differences in fruit and seed development between PP-abscised and non-abscised groups, and the formation of a protective layer at the peduncle RECs were all similarly observed in *P. sargentii* (Fig. 6I–L and Fig. S5).

**Figure 7:**
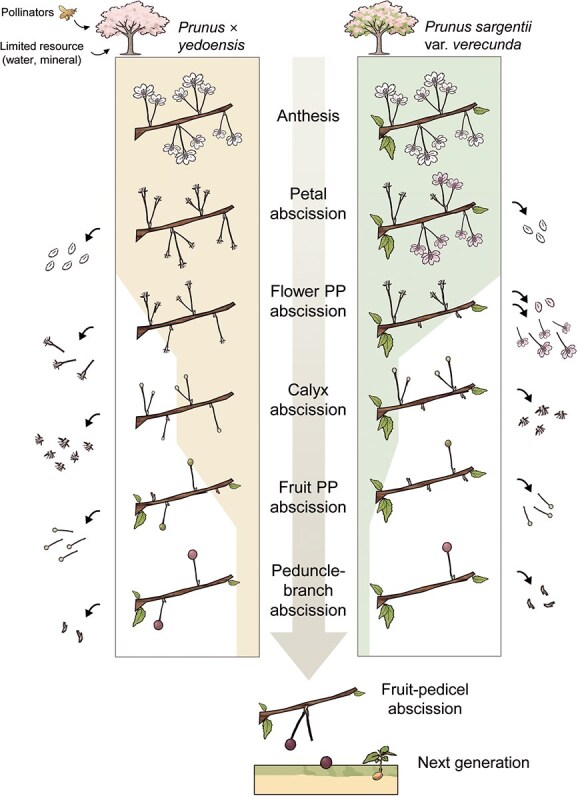
Diagram of the hierarchical abscission program regulating reproductive allocation in *P. yedoensis* and *P. sargentii*. Model illustrating five spatially and temporally distinct abscission events for petals, the calyx, flower PP, fruit PP, and peduncle–branches, during reproductive development in *P. yedoensis* and *P. sargentii*. Each event serves as a developmental gating point that filters reproductive organs based on fertilization status and resource availability. The number of flowers or fruits remaining at each stage, along with the progressively narrowing shaded background, reflects actual retention proportions. In *P. yedoensis*, petal abscission is pollination-independent, whereas petals are retained in unfertilized flowers and shed only upon fertilization in *P. sargentii*. These unfertilized flowers are removed via flower PP abscission, and even fertilized flowers undergo a second filtering step through fruit PP abscission, which eliminates a substantial proportion of developing fruits to adjust final fruit number in line with the plant reproductive capacity.

Together, these findings demonstrate that *P. yedoensis* and *P. sargentii* both employ a hierarchical abscission program that filters reproductive structures in sequence, from petals to flower pedicels to fruit pedicels, to finely tune final fruit load. Although the overall framework is conserved, *P. sargentii* exhibits a distinct early-phase adjustment by delaying petal abscission and favoring flower PP abscission in response to fertilization variability. This species-specific modulation likely reflects an adaptive mechanism to accommodate fluctuating pollination success, enabling both species to avoid resource overinvestment and converge on a reproductively optimal fruit number (Fig. 7).

## Discussion

Among the countless flowers that bloom on a single tree, which ones are ultimately allowed to mature into fruits, and how is this decision made? This question prompted the presentstudy, inspired by the fleeting abundance of cherry blossoms in spring. *Arabidopsis* has been instrumental in revealing the molecular mechanisms of floral organ abscission, but it falls short when trying to capture the complexity of reproductive strategies in long-lived, cross-pollinating perennials, especially woody plants.

In this study, we investigated floral organ abscission in two *Prunus species* and identified five spatially and temporally distinct abscission events: petal, calyx, flower PP, fruit PP, and peduncle–branch abscissions. Rather than serving as mere developmental transitions, these events functioned as hierarchical gating steps that sequentially filtered reproductive structures. Surplus flowers, initially produced to buffer against fertilization failure, were progressively eliminated to adjust the final fruit load in accordance with nutrient availability and pollination efficiency. This stepwise filtering program illustrates how perennial woody plants balance flower overproduction with their selective retention to align reproductive output with both environmental conditions and internal constraints. Our findings provide a conceptual framework for understanding abscission as an active strategy that modulates reproductive investment to optimize efficiency under fluctuating conditions rather than as a passive outcome of development (Fig. 7).

We observed a particularly striking divergence in the pattern of petal abscission between *P. yedoensis* and *P. sargentii*. In *P. yedoensis*, petals were shed shortly after anthesis, independently of fertilization, whereas in *P. sargentii*, petal retention was tightly linked to fertilization. This difference likely reflects broader ecological and physiological strategies*. P. yedoensis* produces a profusion of flowers that bloom before leaf emergence, possibly maximizing pollinator attraction at the cost of limited photosynthetic support. However, the artificial hybrid origin of *P. yedoensis* [55] should also be taken into account, as the influence of human selection—favoring early and profuse flowering in spring—cannot be entirely excluded. A comparison with its parental species would help address this possibility. In *P. sargentii*, flowers appear concurrently with leaf expansion, thereby ensuring a more stable energy supply. Additionally, *P. sargentii* typically inhabits cooler, high-altitude environments where delayed petal abscission may increase the chances of successful pollination [56].

These contrasting patterns raise a broader question: what determines the intrinsic timing of floral organ abscission? We propose that floral abscission operates under an internally governed timing program that unfolds after anthesis. This developmental trajectory likely coordinates multiple steps such as stigma senescence, ovule fate, and petal detachment, in a temporally linked manner. In this framework, fertilization does not act as the primary trigger, but rather as a modulatory input that can accelerate pre-existing developmental transitions. Such a mechanism reconciles the strategies observed in different *Prunus* species: in *P. yedoensis*, the timer proceeds independently of fertilization, whereas fertilization advances timing in *P. sargentii*, ensuring that petals are only shed after reproductive success is confirmed.

This model finds support in *Arabidopsis*, in which floral organ abscission is delayed, but not prevented, in sterile mutants [19, 57–59], suggesting the presence of a time-dependent mechanism. Importantly, abscission still proceeds even in mutants lacking components of the core ethylene signaling pathway [17], indicating that this temporal program operates independently of these canonical regulators. As a self-fertilizing species, *Arabidopsis* has drawn limited attention to fertilization-linked abscission dynamics. Yet this underexplored dimension now deserves closer investigation. Revisiting classic *Arabidopsis* mutants in this light may help uncover the molecular basis of a hidden layer of abscission control: one that, while playing a minor role in *Arabidopsis*, may be more central to shaping reproductive strategies in other species, including *Prunus*.

Another key question concerns spatial specificity: how do five temporally distinct abscission events occur in closely adjacent tissues in *Prunus*, despite the diffusible nature of signals like ethylene and small peptides? Our findings suggest that spatial precision is not governed by ethylene concentration alone, but rather by a combination of tissue-specific responsiveness (i.e. ethylene competence) and localized signaling dynamics, particularly in the PP AZ. These differences in ethylene competence may be linked to auxin signaling. Auxin licenses abscission by conferring ethylene competence, yet elevated auxin attenuates ethylene sensitivity [60]. Auxin synthesized in fertilized fruit could be exported to adjacent tissues [61, 62], where it can pre-pattern—or maintain—domains of differential ethylene competence. Although the concept of ethylene competence was proposed decades ago [63], its cellular basis has remained elusive. We show here that competence correlated with renewed cell division within the AZ. Although the AZ is pre-patterned by anthesis, it remains developmentally quiescent until triggered by internal cues. Upon reactivation, it undergoes cell division, followed by differentiation into RECs and SECs and, in some cases, lignin deposition, suggesting a coordinated developmental program.

Although such REC and SEC specialization was previously described in *Arabidopsis* [7], the upstream regulatory pathway is unknown, and it remains unclear whether renewed cell division is also required to initiate abscission in *Arabidopsis*. In *Prunus*, the clear onset of cell division during AZ activation raises the possibility that the AZ functions as a previously unrecognized, temporally restricted meristem. Its transient and spatially confined reactivation suggests the presence of a developmental niche, potentially governed by meristem maintenance modules akin to those controlling floral meristems. Future studies integrating transcriptomic, epigenomic, and lineage-tracing approaches will be essential to uncover how competence and plasticity are conferred to AZs and whether meristem-like regulatory logics underlie spatial specificity in abscission.

Finally, we observed that fertilization alone does not guarantee fruit retention: >80% of all fertilized flowers were ultimately discarded. This finding indicates the presence of a stringent post-fertilization selection mechanism. In contrast to species such as *Arabidopsis* or *Brassica napus*, in which fruit number is largely determined during floral initiation [64], *Prunus* species can dynamically adjust their fruit number after flowering via sequential abscission events. This mechanism challenges existing notions of a fixed target fruit number and highlights the importance of post-fertilization fruit filtering. Future studies could examine how environmental or nutritional conditions alter the threshold for fruit retention, with direct implications for crop management in species like apple (*Malus domestica*) and pear (*Pyrus* sp.), where excessive fruit set often necessitates manual thinning [65]. Understanding how plants determine and store decisions about fruit load could help inform more efficient agricultural management practices.

From an ecological perspective, although *Prunus* species exhibit the *r*-selected trait (suited to a reproductive strategy of rapid reproduction under high mortality) of copious flowering, the selective removal of underdeveloped fruits reflects a partial shift toward *K*-selection (slower reproduction and greater parental care in the face of competition) [66, 67]. This potential shift aligns with the view that reproductive strategies exist along a continuum rather than as discrete categories [67, 68]. By shedding low-fitness fruits and retaining fully developed, dispersal-competent fruits, *Prunus* species enhance their overall reproductive efficiency and promote effective seed dispersal [69, 70]. These strategies likely evolved to balance the uncertainty of pollination with the high cost of fruit maturation in perennial systems.

Taken together, our findings suggest that organ abscission in perennial woody plant species is not merely a passive developmental conclusion, but an actively regulated program that integrates physiological status, fertilization outcomes, and ecological context. By dissecting these spatially and temporally resolved abscission programs, we begin to uncover the flexible yet finely tuned strategies that underlie reproductive success in long-lived plants. Importantly, our work highlights the critical decision points within the abscission hierarchy, especially the selective retention or elimination of fertilized flowers, as key regulatory nodes where developmental and ecological signals converge. This conceptual framework deepens our understanding of how plants optimize reproductive output and provides a strategic foundation for future studies into the molecular, cellular, and ecological controls of organ shedding in perennial systems.

## Materials and methods

### Plant materials and growth conditions

Four- to 5-year-old *P. yedoensis* trees used for pollination tests and ACC treatments were cultivated in a pollinator-free greenhouse prior to anthesis to ensure controlled experimental conditions. The greenhouse was maintained at a temperature of 20°C–22°C under natural light conditions. Each tree was grown in a 43.5-cm-diameter, 44-cm-tall polyethylene pot containing 35 l of a soil mixture composed of cocopeat, perlite, vermiculite, zeolite, decomposed granite, peat moss, and bark at a volumetric ratio of 65:15:10:5:2:1.5:1.5 (v/v). Watering was carried out three times per week, and no fertilizer was applied. For the remaining experiments conducted under open-field conditions, specimens of *P. yedoensis* and *P. sargentii* aged ≥7 years were collected from Seoul National University (37°27′36″N, 126°57′09″E) and Mt. Gwanak (37°26′44″N, 126°57′49″E), both located in the Gwanak district of Seoul, South Korea [56].

For peptide treatment assay, *A. thaliana* seeds were surface-sterilized, then stratified for 3 days at 4°C in darkness. Seeds were sown on half-strength Murashige and Skoog (MS) medium containing 4.4 g l^−1^ MS salts (Duchefa Farma B.V., Haarlem, The Netherlands), 1% (w/v) sucrose, and 2-(N-morpholino) ethanesulfonic acid (MES), adjusted to pH 5.7 with potassium hydroxide (KOH), and solidified with 0.8% (w/v) agar (Duchefa Farma B.V., Haarlem, The Netherlands). Ten days after sowing, seedlings were transferred to soil. Plants were grown in a controlled-growth chamber under long-day conditions (16 h light at 22°C and 8 h dark at 18°C) with 50% relative humidity.

### Phenotypic assessment

To monitor abscission events in *P. yedoensis* and *P. sargentii*, petal abscission, flower PP abscission, calyx abscission, and fruit PP abscission were scored daily at the branch and peduncle levels from 28 March to 20 June 2025. The onset of each abscission event was recorded, and the number and proportion of each unit, including fully matured fruits, were quantified. Photographs corresponding to each abscission event were also collected using a KCS3-50 camera (Korea Lab Tech, Gyeonggi-do, South Korea) and Leica M205FA stereomicroscope (Leica, Wetzlar, Germany). For seed imaging, the pericarp was manually removed from the fruit. Fruit and seed sizes were measured using ImageJ software [71].

### Transcriptomic analysis

Time-series RNA-seq data obtained from whole flowers (NCBI BioProject Accession: PRJDB12348) were used for the analysis (Table S2) [41]. Raw sequencing reads were quality-filtered using PRINSEQ (v0.20.4.7) [72], and retained reads were mapped to the *P. yedoensis* reference genome (CYE_r3.1.pseudomolecule.fasta.gz from DBcherry; https://cherry.kazusa.or.jp/) with HISAT2 (v2.2.1) [73, 74]. Gene-level abundances were estimated with StringTie (v1.3.4) [75] using the *P. yedoensis* GFF3 annotation (CYE_r3.1.pseudomolecule.genes.gff.gz from DBcherry, https://cherry.kazusa.or.jp/) [74], transcript-per-million (TPM) estimates were compiled into a gene-by-sample expression matrix. Between-sample compositional bias was corrected by trimmed mean of M-values (TMM) normalization implemented in edgeR (v3.19) in R [76]. For gene annotation, BlastP searches were performed against the TAIR10 *A. thaliana* protein database (Araport11_pep_20250411.gz from The Arabidopsis Information Resource; https://www.arabidopsis.org/), with an e-value cut-off of 0.01 [77]. For the pre-defined gene set, row-wise *z*-scores were computed and visualized as a heatmap using the pheatmap package (v1.0.12) in R [78].

### Multiple sequence alignment, phylogenetic analysis, and protein structure prediction

Multiple sequence alignment was performed using ClustalW, and the alignment results were visualized with CLUSTAL Omega (v.1.2.4) [79]. Phylogenetic analyses were conducted using MEGA (v.12) [80]. An ML method was applied with the Jones–Taylor–Thornton substitution model for amino acid sequences. Rate variation among sites was modeled using a gamma distribution with five discrete categories. Branch support was evaluated with a standard bootstrap analysis of 4000 replicates. Alignment gaps and missing data were treated with the partial deletion option and a 95% site coverage cut-off. For ML heuristic searches, the nearest-neighbor-interchange algorithm was employed, and the initial tree for ML inference was generated automatically using the NJ and maximum parsimony methods.

Protein structure prediction was performed using Omegafold with default parameters [81]. Prediction confidence was assessed using predicted local-distance difference test (pLDDT) scores, and the resulting structures were visualized with ChimeraX (v. 1.10.1) [82].

### Peptide treatment assay

Wild-type *A. thaliana* flowers at Stage 14 were collected from primary inflorescences. Each flower was excised together with short segments of the stem and its pedicel to generate stem–pedicel–flower explants, which were placed on half-strength MS medium. Media were supplemented with either 10 μM mAtIDA (GVPIPPSAPSKRHN) or 10 μM mPyIDA (GTPIPPSGPSKRHN), both dissolved in distilled water; control media were supplemented with an equal volume of distilled water. Plates were incubated in growth chambers under long-day conditions (16 h light at 22°C and 8 h dark at 18°C) at 50% relative humidity. All peptides were synthesized by COSMOGENTCH at >95% purity.

### Pollination treatment

For pollination treatment, *P. yedoensis* trees grown in a pollinator-free greenhouse were used. At 0–1 DPA, a subset of flowers corresponding to 0%, 25%, 50%, or 75% of the total number of flowers per tree, or all (100%) flowers were marked and subsequently hand-pollinated. Since *P. yedoensis* is self-incompatible, compatible pollen was obtained from five donor trees. Donor trees were spatially separated to prevent unintended pollination, while growth conditions were kept identical. Anthers were collected at 0 DPA, and pollen grains were released by inducing mechanical dehiscence of the anthers. The released pollen was then applied to the marked flowers.

### Scanning electron microscopy

Samples were fixed in 2.5% (v/v) glutaraldehyde in 0.1 M sodium phosphate buffer (pH 7.2), followed by vacuum infiltration for 1 h at room temperature. After the vacuum was released, samples were incubated overnight at room temperature. Subsequently, samples were washed three times in 1× phosphate-buffered saline (PBS) for 20 min each time and incubated in 1% (v/v) Triton X-100 at room temperature for 3 days. Post-incubation, samples were transferred to 1% (w/v) osmium tetroxide in 1× PBS and incubated overnight in the dark at 4°C. Samples were then washed three times in 1× PBS for 20 min each time and then subjected to ethanol dehydration using a graded series of ethanol solutions (30%, 50%, 60%, 70%, 80%, 90%, 95%, all v/v, and three times in 100%). Each step was conducted for a minimum of 2 h, with the final 100% ethanol step performed overnight. To maintain structural integrity, tissue specimens were dehydrated using a critical point dryer (EM CPD300, Leica, Vienna, Austria). The dried samples were affixed onto metal stubs with conductive carbon tape and coated with a thin platinum layer using a sputter coater (EM ACE200, Leica, Vienna, Austria) to improve electrical conductivity and imaging clarity. The prepared samples were then examined under high-vacuum conditions using a scanning electron microscope (JSM 6390LV, JEOL, Tokyo, Japan).

### ACC application via lanolin paste

ACC was freshly dissolved in distilled water to a concentration of 1 mM, then incorporated into melted lanolin (pre-warmed to 60°C) to yield a final concentration of 100 μM. A thin layer of the resulting lanolin paste was applied to specific floral organs, which were subsequently covered with plastic wrap to maintain contact: the calyx base for petal abscission treatment, and both the peduncle and pedicel sides for flower PP abscission treatment. Control paste contained an equivalent volume of dimethyl sulfoxide (DMSO). All experiments were conducted in a greenhouse, and control and ACC treatments were applied to separate trees to prevent broad ACC- and ethylene-mediated effects.

### Resin sections for histological examination

Tissue samples were fixed overnight in 4% (v/v) glutaraldehyde (Sigma-Aldrich, Missouri, USA) prepared in 1× PBS (Bio-Rad Laboratories, California, USA). Following fixation, samples underwent dehydration through a graded ethanol series (30%, 40%, 50%, 60%, 70%, 80%, 90%, 95%, and 100%, all v/v). Infiltration with resin was carried out by gradually increasing the concentration of Technovit 7100 resin (Kulzer Technik, Hanau, Germany) in ethanol through a stepwise dilution series (1:5, 2:5, 3:5, 4:5, 5:5, 3.5:2.5, 4.5:2.5, 5.5:2.5, 6.5:2.5, 7.5:2.5, v/v), with each infiltration step conducted for 2 h. Fully infiltrated samples were embedded in resin, and 4-μm-thick sections were cut using a rotary microtome (Histocore AUTOCUT, Leica, Vienna, Austria). The sections were stained with 0.01% (w/v) toluidine blue (Thermo Fisher Scientific, Massachusetts, USA), and images were acquired using an Axioscope 5 microscope (Zeiss, Jena, Germany).

### DAB staining for hydrogen peroxide detection

Hydrogen peroxide accumulation was visualized by staining samples with 3,3′-diaminobenzidine (DAB; Sigma-Aldrich, Missouri, USA) [83]. Vacuum infiltration was carried out using a 0.1% (w/v) DAB solution prepared in distilled water and adjusted to pH 5.8 with KOH. Samples were incubated in the dark for 6 h to allow for DAB staining. After staining, tissues were fixed and chlorophylls were removed by incubating samples in a bleaching solution (ethanol:lactic acid:glycerol = 3:1:1, v/v/v) at 70°C until complete decolorization was achieved. For petal AZs, samples were incubated in the bleaching solution at 70°C for 1 h. In the case of PP and peduncle–branch AZs, the same 1-h incubation at 70°C was performed four times with fresh bleaching solution exchanged after each step. Samples were mounted in bleaching solution and imaged using a Leica M205FA stereomicroscope (Leica, Wetzlar, Germany).

### Cytosolic pH visualization using BCECF

A 10-μM solution of BCECF-AM (Invitrogen, Massachusetts, USA), prepared from a 10-mM stock dissolved in DMSO (Sigma-Aldrich, Missouri, USA) and diluted in 1× PBS (Bio-Rad Laboratories, California, USA), was applied to the surface of tissue samples [28]. The samples were incubated in the dark for 30 min to allow dye uptake and then washed four times with 1× PBS to remove unincorporated dye. Fluorescence imaging was performed using an LSM 900 confocal microscope (Zeiss, Jena, Germany) with excitation at 488 nm and emission detection in the 505–525 nm range.

### Phloroglucinol–HCl staining for lignin detection

To visualize lignin accumulation, AZs from petals, PPs, and calyces were incubated in 3% (w/v) phloroglucinol in absolute ethanol at room temperature for 1 week, followed by incubation at 37°C for an additional 3 weeks in a controlled chamber [84]. After incubation, 37% hydrochloric acid (HCl; Samchun Chemical Co., Ltd., Gyeonggi-do, South Korea) was applied to the tissues to initiate the staining reaction, with petals incubated for 1 min and PP and calyx tissues incubated for 10 min. Samples were subsequently mounted in either 37% HCl or ethanol, and images were acquired using a Leica M205FA stereomicroscope (Leica, Wetzlar, Germany).

### Permeability assay using toluidine blue staining

To assess permeability, PP and peduncle–branch RECs were immersed for 10 min in an aqueous solution containing 0.05% (w/v) toluidine blue O (Thermo Fisher Scientific, Massachusetts, USA) and 0.01% (v/v) Tween-20 (Sigma-Aldrich, Missouri, USA) [85]. After staining, samples were rinsed twice with distilled water to remove excess dye. Images were acquired using a Leica M205FA stereomicroscope (Leica, Wetzlar, Germany).

### Petal color acquisition, correction, and visualization

To characterize petal coloration across developmental stages, from anthesis and petal abscission to flower PP abscission, each sample was photographed using a KCS3–50 camera (Korea Lab Tech, Gyeonggi-do, South Korea) under standardized illumination within a matte black chamber. A color reference strip containing six pre-defined patches was included in each image for calibration. All image processing was conducted in MATLAB R2023b (MathWorks, Natick, MA, USA) using the Image Processing Toolbox. Reference regions were manually selected per image, and their red–green–blue (RGB) pixel values were converted into N × 3 matrices to compute patch-wise mean values. A 3 × 3 linear correction matrix was then estimated via least-squares regression to map the measured RGB values to a pre-defined target set and applied to the entire image [86]. Corrected images were clipped to the 0–255 range, converted to 8-bit unsigned integers, and used for subsequent analysis.

To represent petal patterns in a compact and interpretable format, dominant colors were extracted from each image based on pixel frequency. Each image was smoothed using a Gaussian filter (σ = 1) to reduce noise. Thirty polygonal regions of interest (ROIs) were manually delineated per image to encompass intact petal areas while excluding regions in shadow or damaged regions. Binary masks were generated to isolate relevant pixels in each ROI. Extracted RGB values were converted to hue–saturation–value (HSV) color space, and pixels with V <0.5 were excluded to minimize background interference. The frequency of the remaining RGB triplet values was computed, and the top 20 most frequent colors were selected for each ROI. From these, a representative set of 400 dominant RGB values was compiled across the 30 ROIs. Using the conversion values for RGB to HSV, all colors were sorted by H to enhance perceptual coherence [87]. The resulting sorted colors were visualized as a horizontal color bar placed beneath each corresponding petal image, enabling intuitive stage-wise comparison of observed petal color patterns [88].

### Statistical analysis

All statistical analyses were carried out using GraphPad Prism software (version 10.5.0; GraphPad Software, https://www.graphpad.com/). Details regarding the number of technical and biological replicates and the specific statistical methods applied are included in the respective figure legends.

## Supplementary Material

Web_Material_uhaf317

## Data Availability

The source code for petal color acquisition, correction, and visualization is publicly available at GitHub: https://github.com/AhyeonChn/-Petal-color-acquisition-correction-visualization.
