# Supplementary material for: A hierarchical abscission program regulates reproductive allocation in Prunus × yedoensis and Prunus sargentii
Source: Hortic Res. 2025 Nov 14;13(2):uhaf317. doi: 10.1093/hr/uhaf317 (PMC12946680; doi:10.1093/hr/uhaf317)
Supplement: Web_Material_uhaf317 [file web_material_uhaf317.zip › Supplementary Figure.pdf]

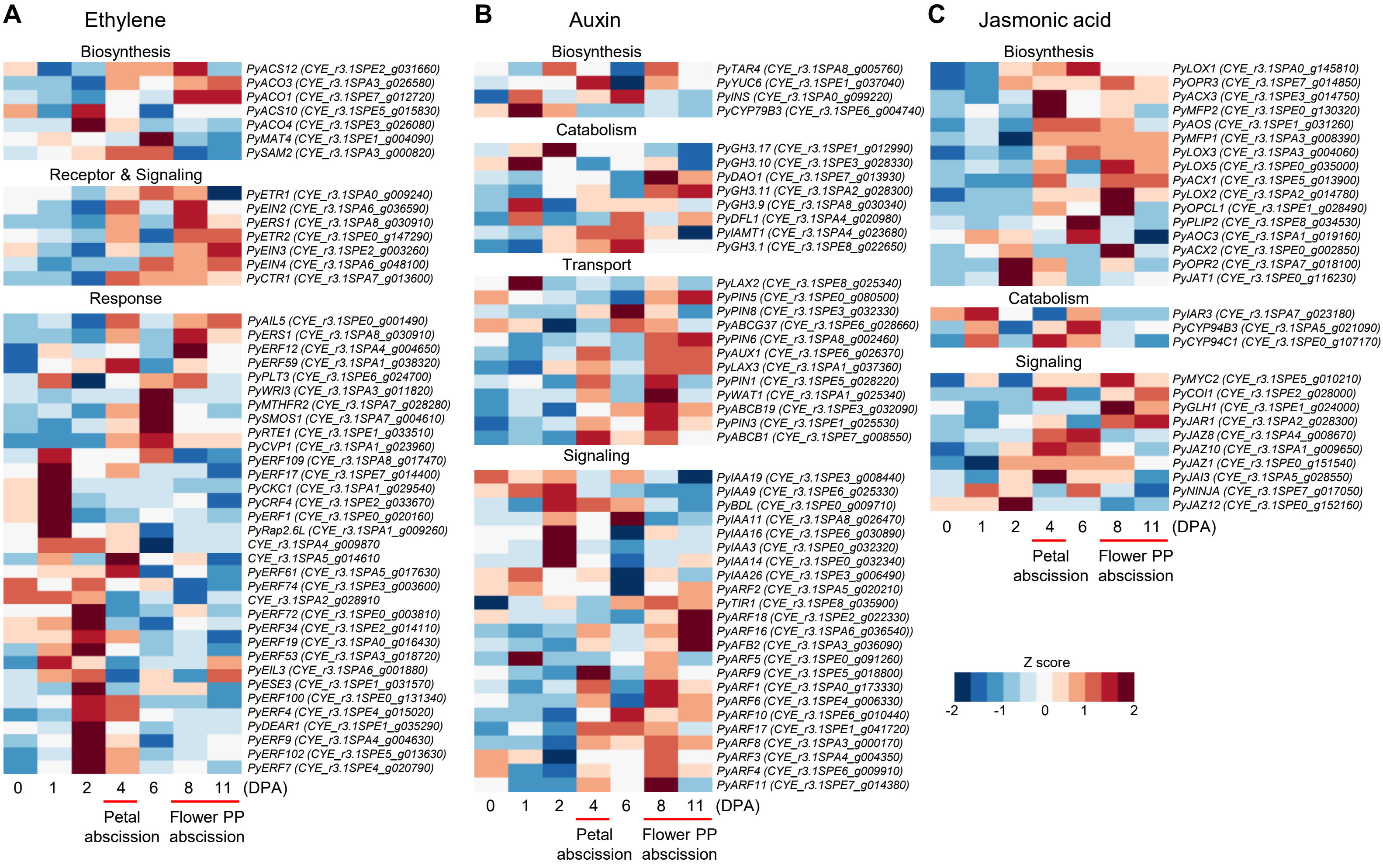

Figure S1. Transcriptomic analysis of hormone-related genes in *Prunus yedoensis*. Heat maps depict expression dynamics of genes associated with biosynthesis, catabolism, transport, receptor, signaling, and response for ethylene (A), auxin (B), and jasmonic acid (C). Gene lists are provided in Supplementary Table S4.

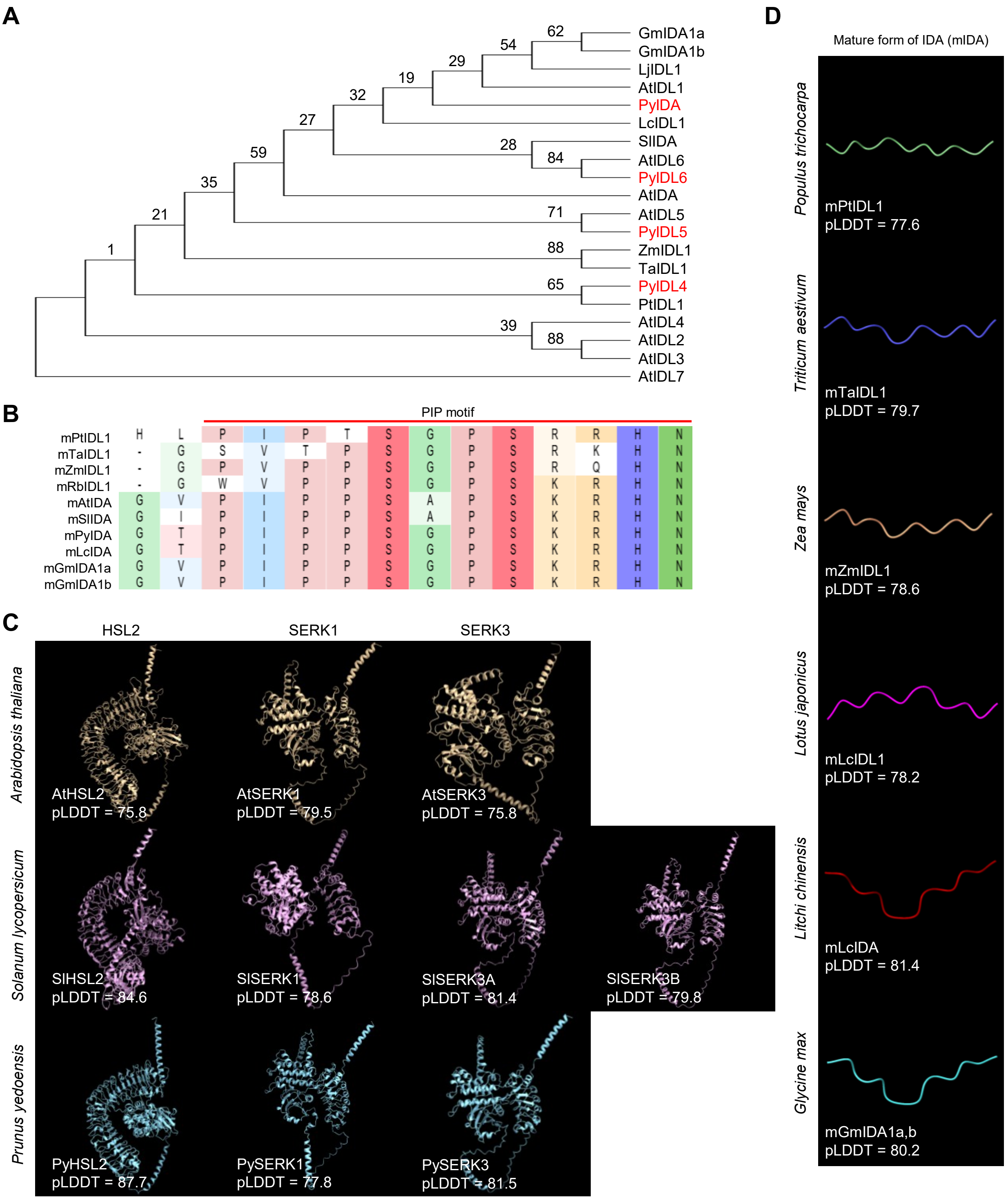

Figure S2. Comparative analysis of IDA/IDLs peptides across multiple species. (A) Maximum-likelihood phylogenetic tree constructed from the whole protein sequence alignment of IDA/IDLs. Branch support values (bootstrap) are indicated at each node. (B) Multiple sequence alignment of IDA peptides from diverse species, aligned against the mature IDA peptide of *Arabidopsis thaliana*. (C) Predicted protein structures of HSL2, SERK1, and SERK3 with confidence values shown as predicted local-distance difference test (pLDDT) scores. (D) Predicted protein structures of mature IDA/IDLs, with confidence values indicated by pLDDT scores.

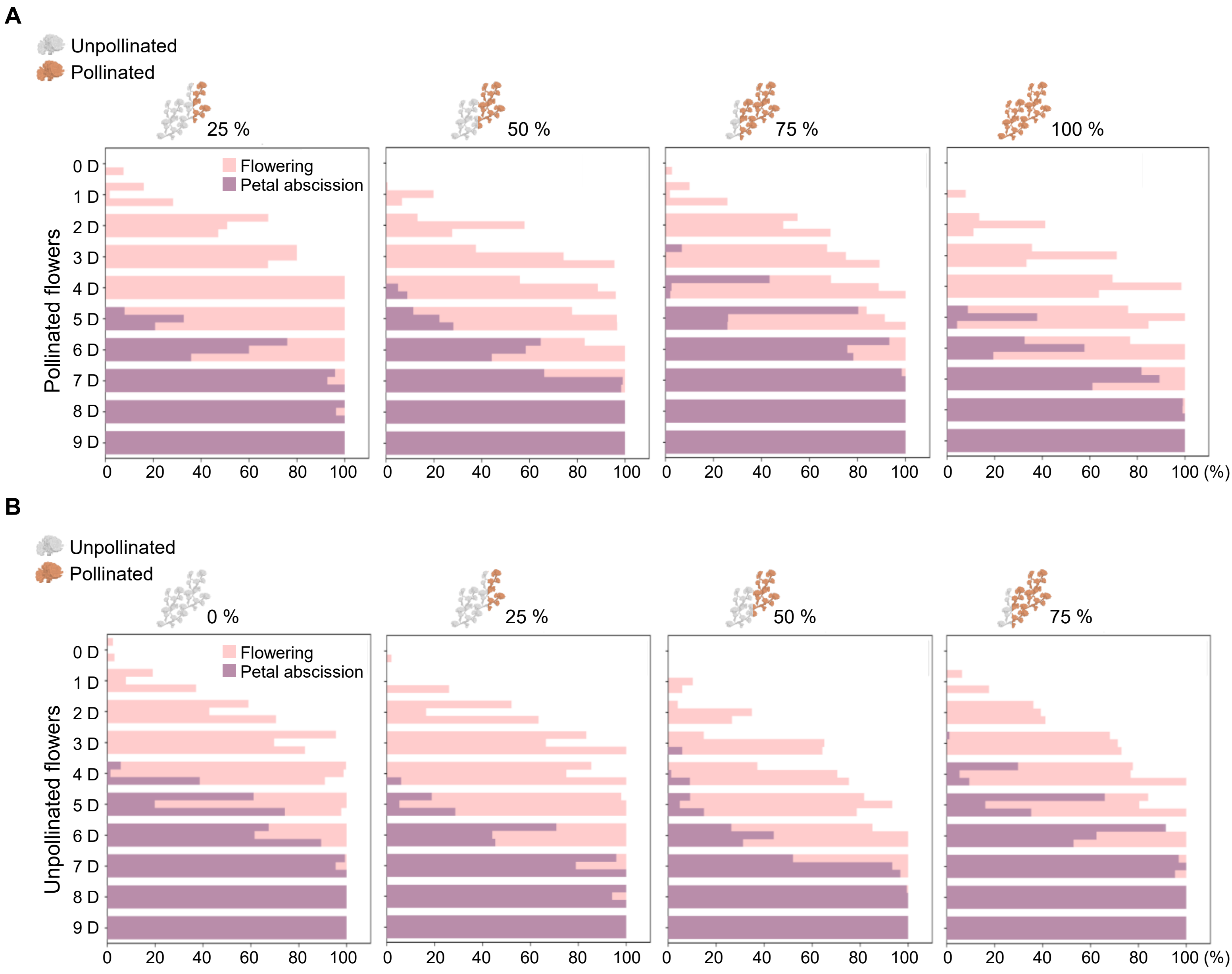

Figure S3. Petal abscission is activated independently of pollination in *Prunus yedoensis*. Petal abscission kinetics were assessed under varying levels of hand pollination; results are presented separately for pollinated (A) and nonpollinated flowers (B). Cross-pollination was performed on 0%, 25%, 50%, 75%, and 100% of all flowers per tree at 0–1 day post-anthesis (DPA). Each horizontal bar represents data from an individual tree (n = 3 trees).

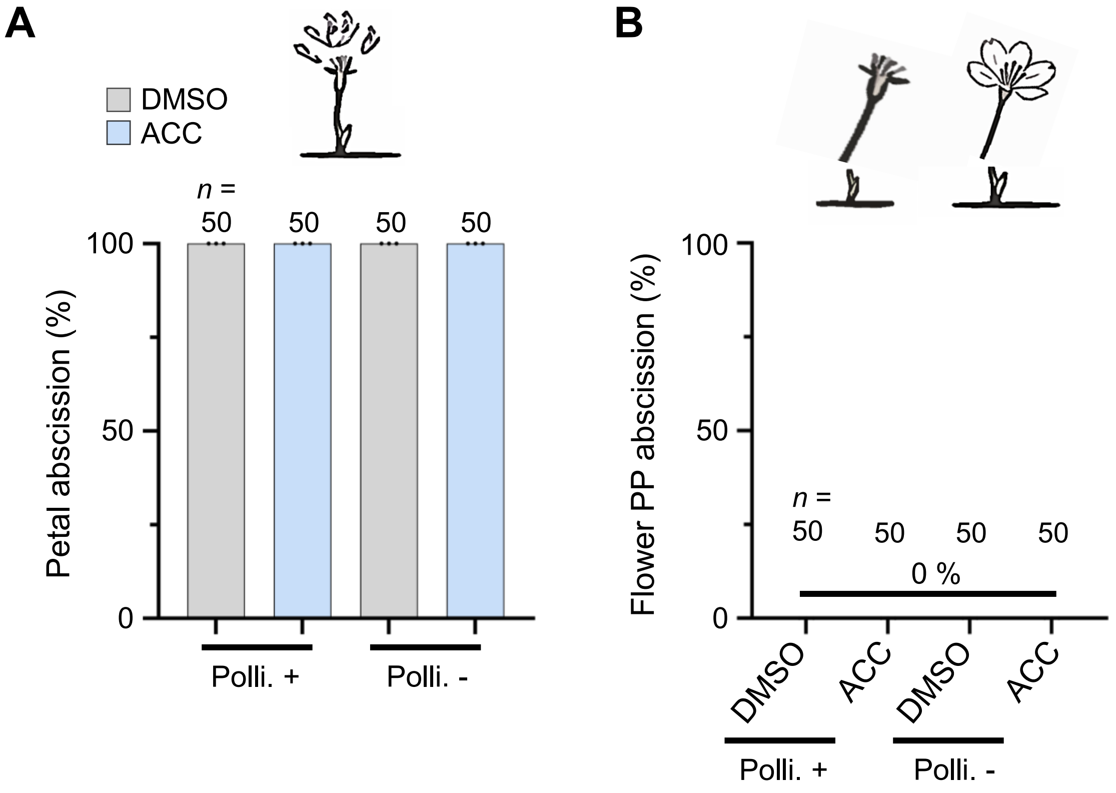

Figure S4. Effects of ACC treatment on petal and flower pedicel–peduncle abscission in *Prunus yedoensis*. Frequency of petal abscission (A) or flower pedicel–peduncle (PP) abscission (B), assessed in *P. yedoensis* following treatment with 100 µM 1-aminocyclopropane-1-carboxylic acid (ACC) or a mock solution (DMSO). Treatments were applied at 1 day post-anthesis (DPA), and abscission was monitored daily; abscission rates at 6 DPA are shown here, prior to the onset of natural flower PP abscission (n = 50 flowers and n = 50 flower pedicels).

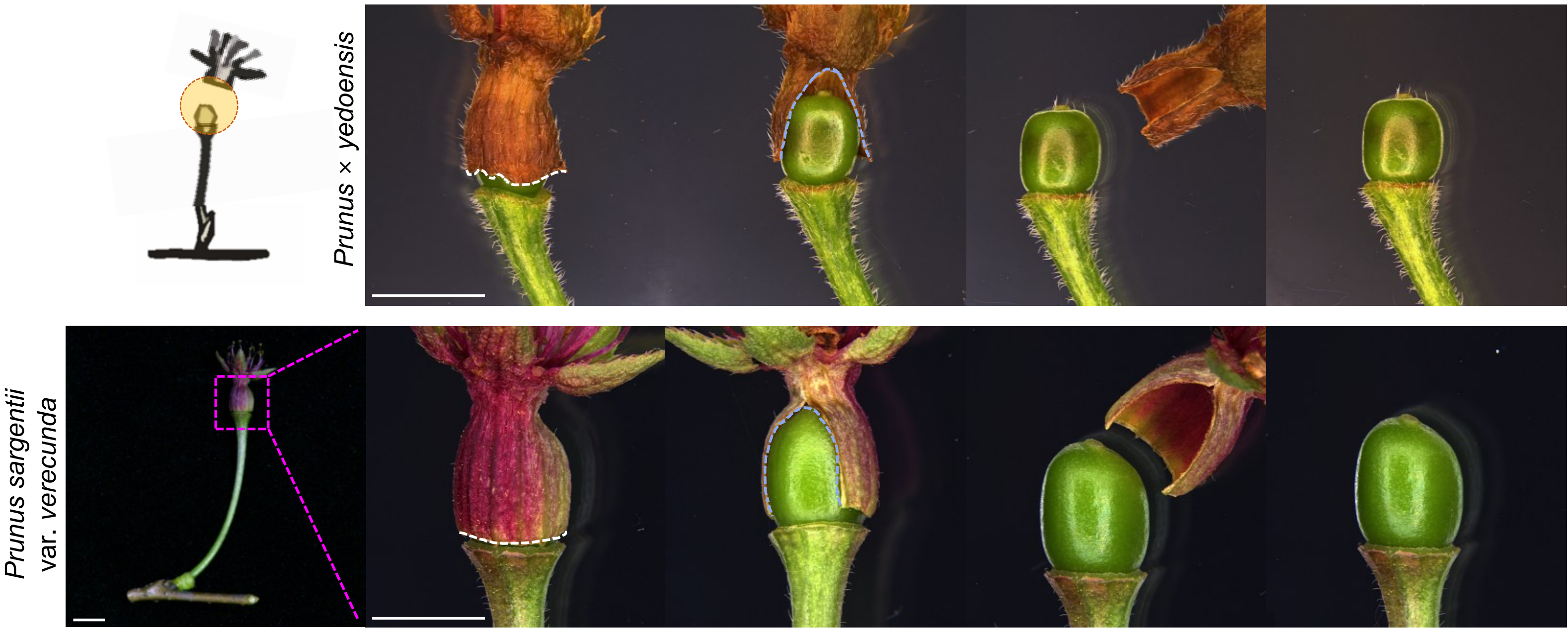

Figure S5. Progression of calyx abscission in *Prunus* species. Representative microscopy images illustrating calyx abscission. In *Prunus* species, a distinct vertical abscission line (white dotted lines) becomes visible on the calyx prior to fruit expansion, whereas a longitudinal abscission line (blue dotted lines) is not visible at this stage. As the fruit expands, the vertical line ruptures first, followed by the longitudinal line. Notably, the longitudinal line appears to form along a predetermined path, as it consistently manifests as a single cleavage site rather than as multiple lines, despite its initial lack of visibility. Ultimately, once longitudinal abscission progresses as the fruit expands, the fruit becomes exposed. The magenta box indicates the region that is shown at higher magnification to the right. Scale bars, 5 mm.

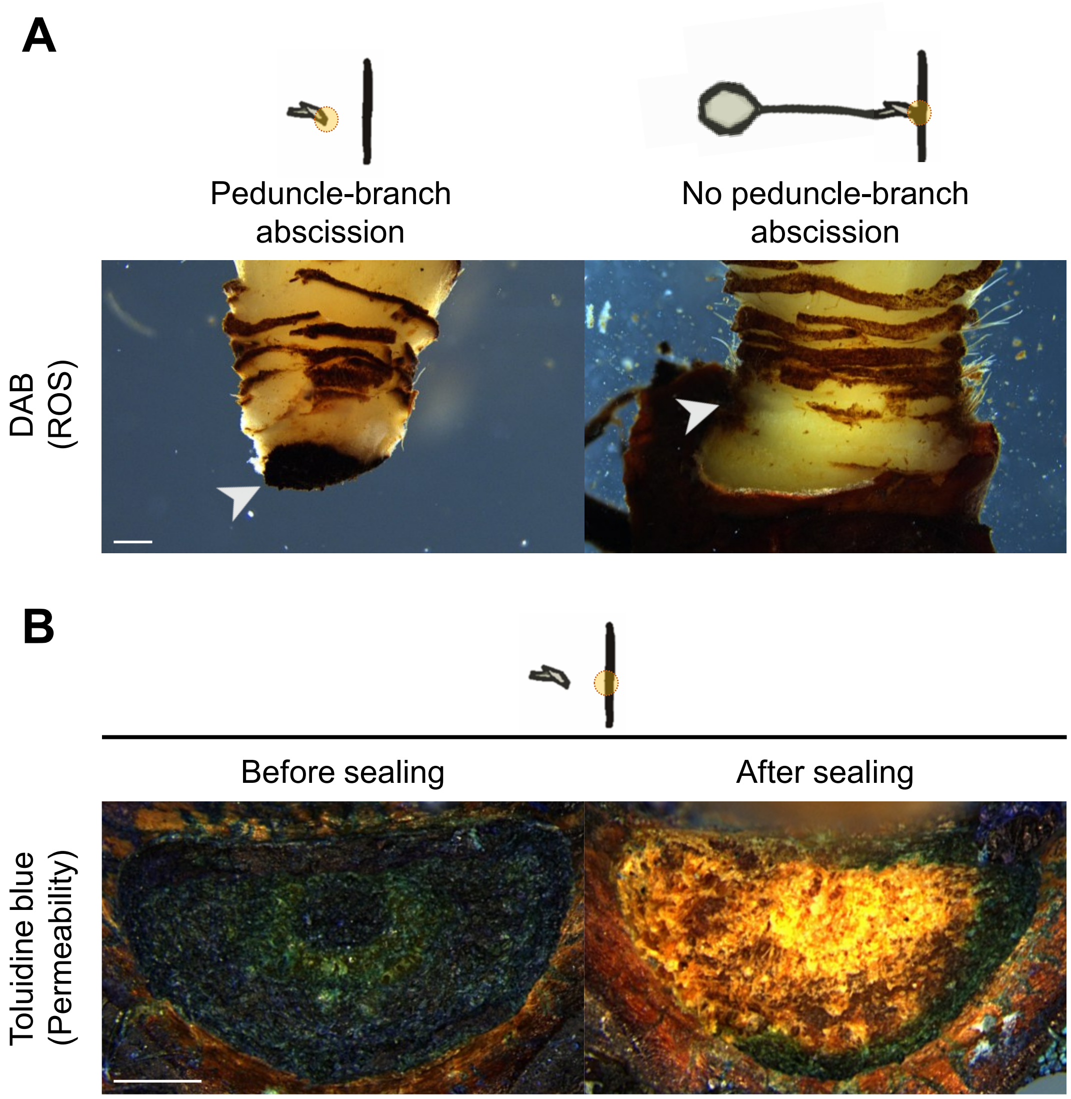

Figure S6. Peduncle–branch abscission in *Prunus yedoensis*. (A) DAB staining to detect ROS accumulation in secession cells at the peduncle–branch abscission zone during abscission at 30 DPA. The white arrowheads indicate the location of the abscission zone (*n* = 15). (B) Toluidine blue staining-based permeability assay to evaluate the formation of a protective layer in residuum cells immediately after peduncle–branch abscission (30 DPA; Before sealing) and at a later time point (40 DPA; After sealing) (*n* = 15). Scale bars, 500 µm.

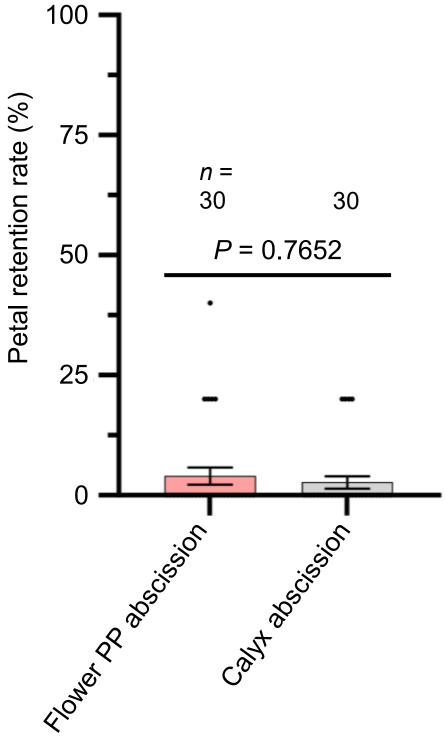

Figure S7. Petal abscission precedes flower pedicel–peduncle abscission and calyx abscission in *Prunus yedoensis*. The proportion of petals remaining was assessed in cases of flower PP abscission or calyx abscission (*n* = 30 flowers). A Mann–Whitney test revealed no statistically significant difference between the two groups (two-tailed P-value = 0.7652). Data are shown as means ± SD.

A

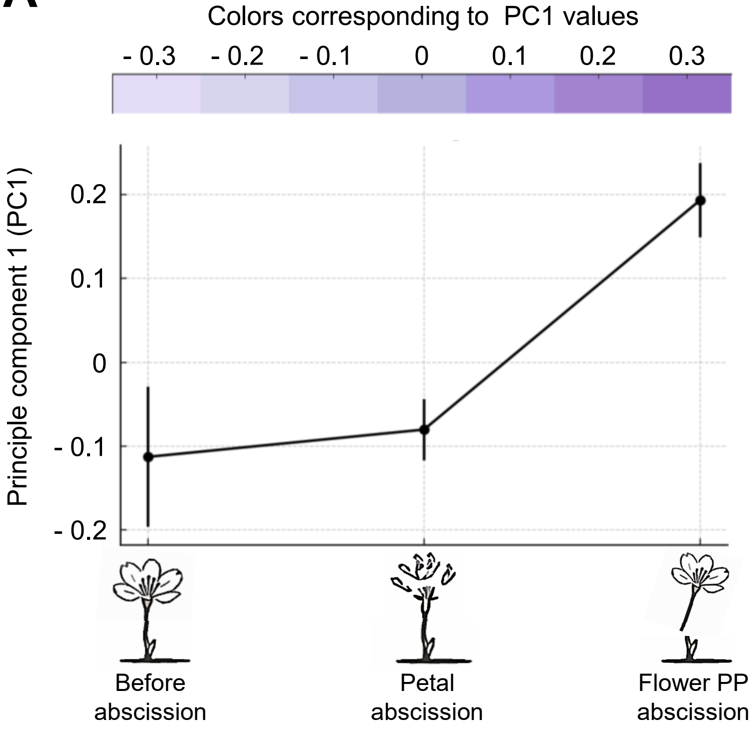

B

Type	L*	a*	b*	C*
Before abscission	46.83 ± 2.65	34.71 ± 5.07	5.16 ± 2.59	35.15 ± 4.86
Petal abscission	43.89 ± 3.00	41.34 ± 4.72	2.41 ± 2.36	41.42 ± 4.77
Flower PP abscission	38.85 ± 2.92	48.43 ± 5.18	0.79 ± 2.52	48.48 ± 5.16

Figure S8. Linearized color progression for petals at different abscission phases in *Prunus sargentii*. (A) Principal component analysis was performed on RGB (red, green, blue) values extracted from petal images at three abscission stages: before abscission, petal abscission, and flower pedicel–peduncle (PP) abscission (*n* = 20 flowers). PC1 values were plotted as mean ± SD. (B) Color parameters were calculated based on the CIELAB color space defined by the International Commission on Illumination: (L*: Lightness, a*: redness (+) to greenness (−), b*: yellowness (+) to blueness (−), c*: chroma, saturation of the color). Each value is shown as mean ± SD.
